# Pregnancy, delivery, and breastfeeding after total gastrectomy for gastric cancer: a case report

**DOI:** 10.1186/s12957-018-1531-2

**Published:** 2018-11-29

**Authors:** Kazuya Higashizono, Sachiyo Nomura, Koichi Yagi, Susumu Aikou, Masato Nishida, Hiroharu Yamashita, Yasuyuki Seto

**Affiliations:** Department of Gastrointestinal Surgery, Graduate School of Medicine, The University of Tokyo, 7-3-1 Hongo, Bunkyo-ku, Tokyo, 113-8655 Japan

**Keywords:** Total gastrectomy, Gastric cancer, Pregnancy and delivery, Breastfeeding, Pregnant after gastrectomy

## Abstract

**Background:**

The reports of pregnancy after total gastrectomy for gastric cancer are rare.

**Case presentation:**

We report a case of a 35-year-old woman, gravida 0, para 0, who became pregnant and delivered a baby 2 years and 6 months after laparoscopic-assisted total gastrectomy for early gastric cancer. Postoperatively, she showed a good progress during the follow-up and was continuously taking oral iron supplement and administered with methylcobalamin intramuscular injection. Two years after gastrectomy, she became pregnant. During the pregnancy, she kept taking iron and vitamin B_12_ supplementation and had a good course of pregnancy and a normal delivery. However, 2 months after the delivery, liver dysfunction was detected via blood examination. The patient switched from exclusive breastfeeding to combined feeding with formula, and her laboratory results returned to normal. During 10 years of follow-up after the delivery, the patient was in good condition without any recurrence and nutritional deficiencies, and her child had thrived.

**Conclusions:**

Careful monitoring and management of iron and vitamin deficiencies are essential during pregnancy and the lactation periods for patients who previously underwent total gastrectomy. During the lactation period, a combination of formula and breastfeeding provides maternal and fetal nutritional support.

## Background

With increased early detection of operable gastric cancers via endoscopic screening and a rising trend in advanced maternal age, it is more common that childbearing women undergo gastrectomy for gastric cancer before pregnancy and delivery [1, 2]. Postoperative gastric cancer patients who are pregnant or have delivered a baby often have trouble with malnutrition due to gastrectomy, despite increasing energy needs and the risk of cancer recurrence [3, 4]. However, there are few studies that report the process of pregnancy and delivery of gastric cancer patients after gastrectomy. We report a case of successful pregnancy and delivery in a patient who previously underwent total gastrectomy for gastric cancer.

## Case presentation

**Fig. 1:**
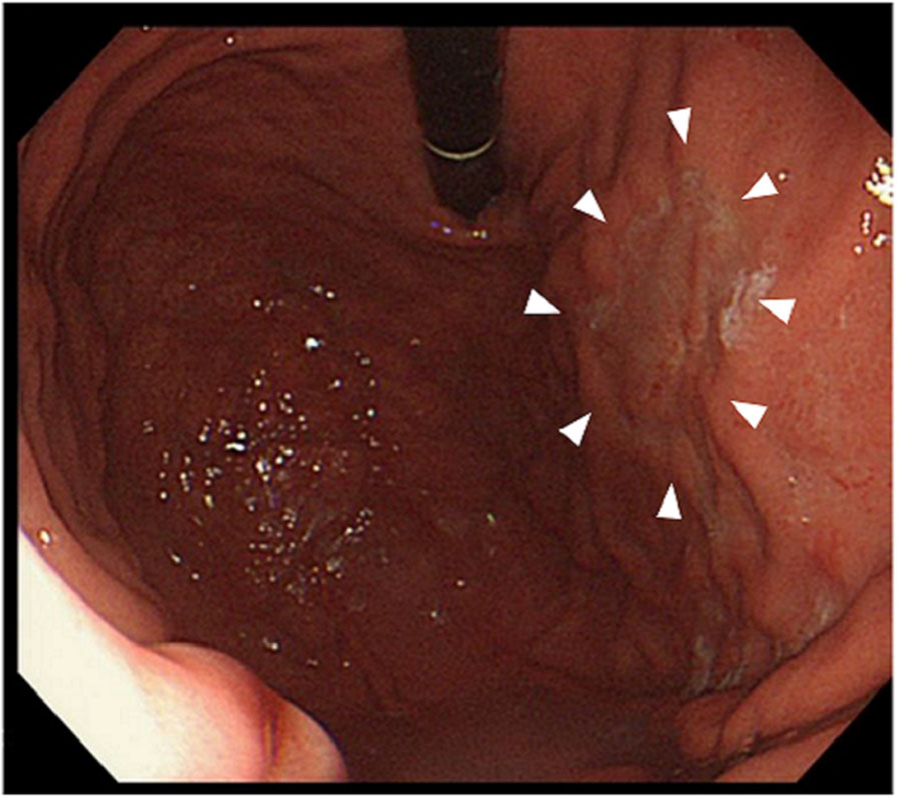
The endoscopic appearance of the lesion: a 30-mm flat and depressed area with smooth tapering of converging folds was observed in the anterior wall of the upper gastric body

**Fig. 2:**
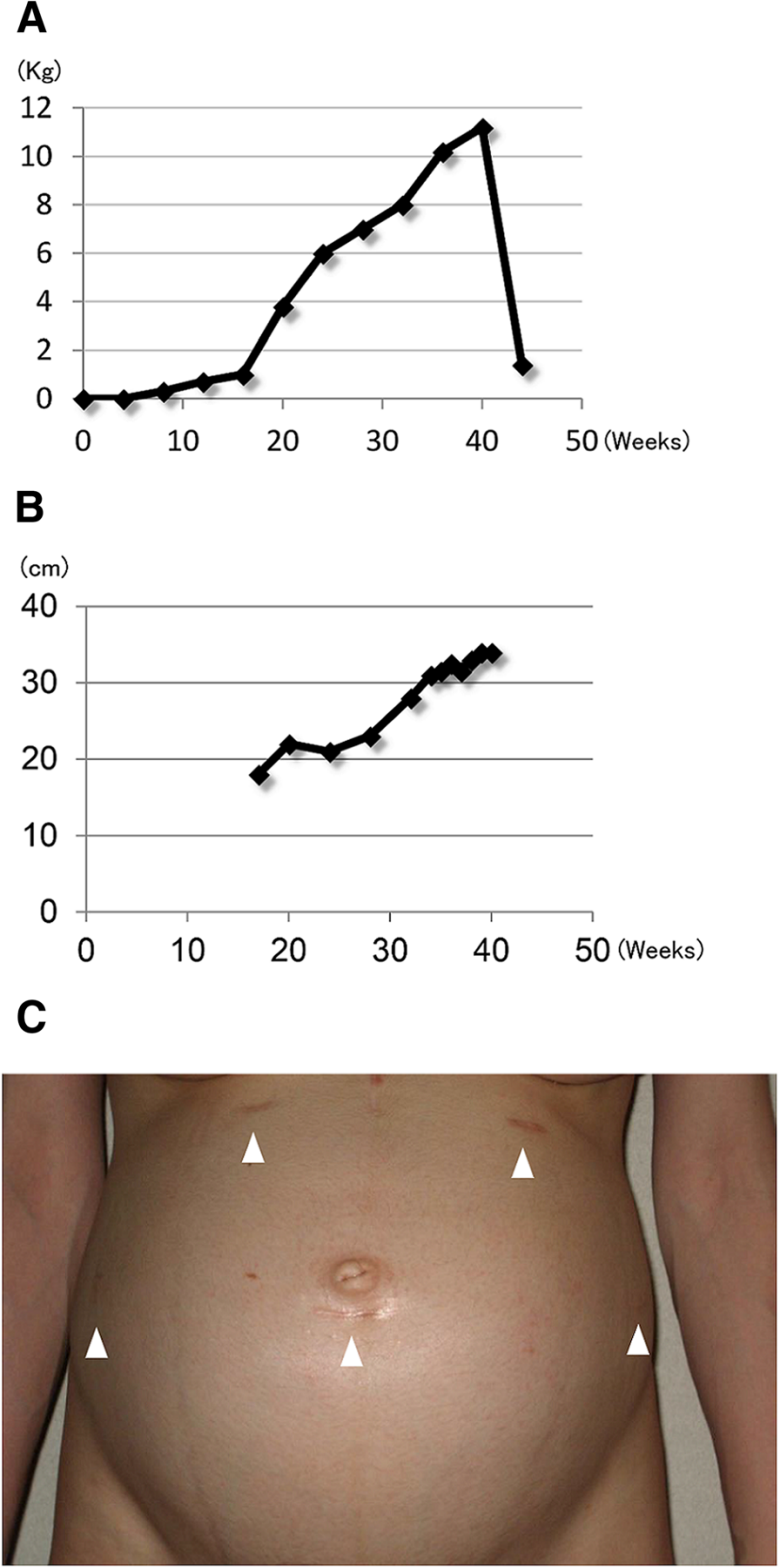
**a** Weight gain of the mother in the course of pregnancy. **b** Uterus height change in the course of pregnancy. **c** The surgical scar sites in the abdomen at 30 weeks of pregnancy

A 35-year-old woman was referred to the Department of Gastrointestinal Surgery of The University of Tokyo Hospital for evaluation of a gastric lesion in August 2005. During esophagogastroduodenoscopy, a 30-mm flat and depressed lesion was identified at the anterior wall of the upper gastric body and was diagnosed as Type 0-IIc gastric cancer (Fig. 1). A pathological analysis of the biopsy specimen determined a moderately differentiated tubular adenocarcinoma (tub2). The patient underwent laparoscopic-assisted total gastrectomy (LATG) with Roux en-Y reconstruction and lymph node dissection (D1 + No. 7) in accordance with the Japanese Gastric Cancer Treatment Guidelines [5]. A pathological analysis of the specimen revealed the depth of the lesion remaining in mucosal layer, without any lymph node metastasis. The patient showed good progress after the operation and did not receive any further treatment including chemotherapy. She was only instructed to continue oral iron supplementation for postoperative chronic anemia after discharge. She became pregnant 1 year and 8 months after the operation. After her pregnancy, radiographic examinations were excluded during the follow-up period while oral iron supplements were continued. In addition, 500 μg of methylcobalamin (MeCbl), an active form of vitamin B_12_, was intramuscularly injected once a month for a potential vitamin B_12_ deficiency, although her serum vitamin B_12_ level was within normal range. No pregnancy-associated discomfort or illness was observed including hyperemesis gravidarum during her pregnancy while the fetus was developing normally. In May 2008, the baby was born by vaginal delivery at 41 weeks of gestation. Delivery time was 4 h and 20 min, and the baby was delivered as a healthy normal child weighing 3076 g. The mother’s weight change and the uterus height change of the fetus are shown in Fig. 2a–c.

**Fig. 3:**
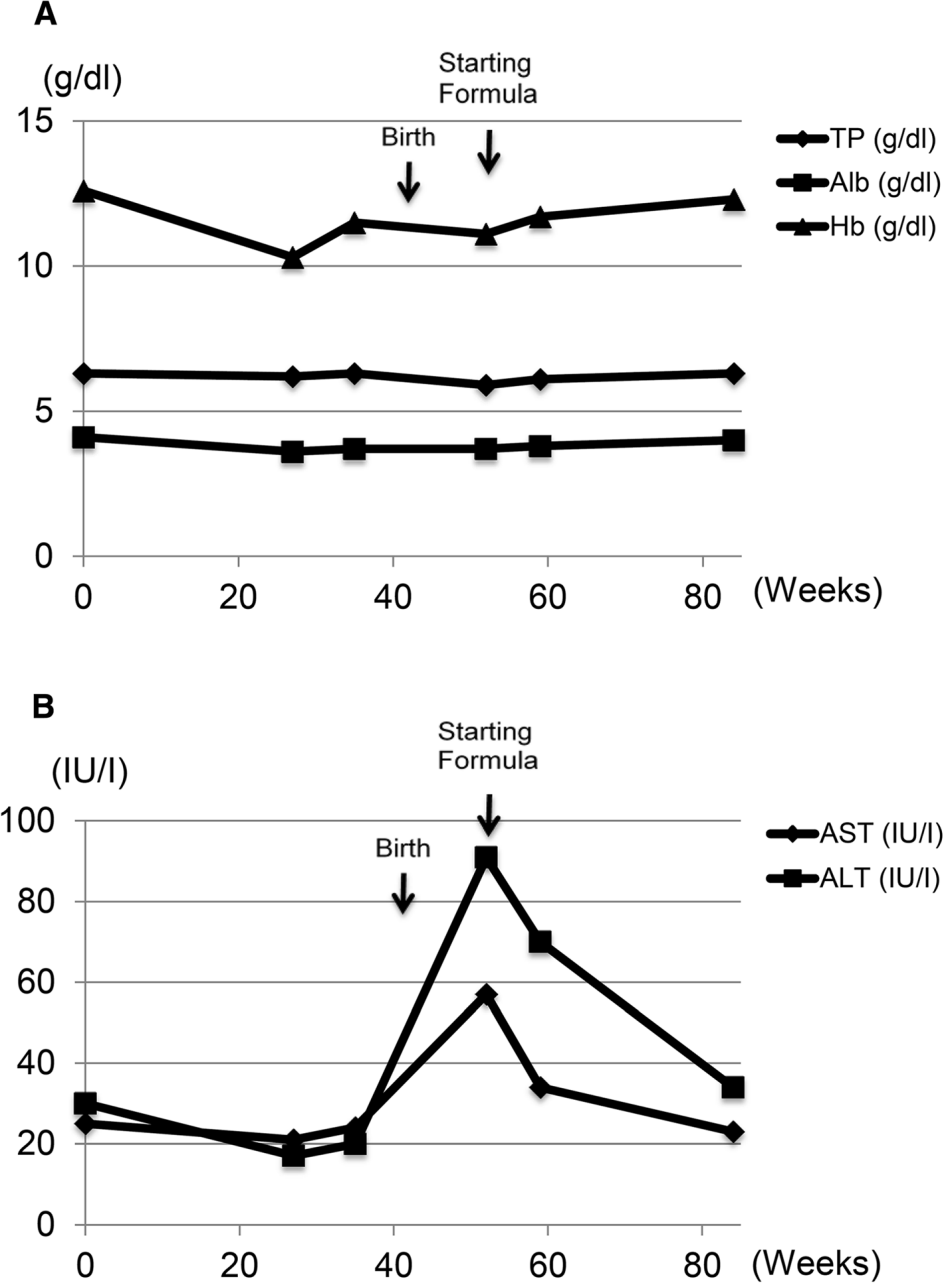
**a** Changes of total protein (TP), albumin (Alb), and hemoglobin (Hb) during pregnancy and the lactation period. TP and Alb were slightly decreased after delivery, but recovered after starting formula. **b** Changes of the serum AST and ALT levels in the mother during pregnancy and the lactation period. They raised after delivery, but returned to normal after starting formula

After delivery, she continued to take oral iron supplements and vitamin B_12_ intramuscular injection and had kept exclusively breastfeeding without any problems until her first postpartum check-up. However, during the blood examination after her postoperative follow-up, 2 months after her delivery, the serum AST (aspartate transaminase) and ALT (alanine transaminase) levels of the patient were found to be elevated. We presumed that excessive oral food intake influenced gastrointestinal absorption leading to liver dysfunction. After changing exclusively breastfeeding into the combination of formula and breastfeeding, the serum AST and ALT levels were restored within the normal range (Fig. 3a, b). The patient has been followed-up for 10 years and has not shown recurrence of gastric cancer. Also, the child showed a good progress.

## Discussion

The relationship between pregnancy and the prognosis of gastric cancer remains controversial; however, some literature reported that pregnancy and/or delivery in young women accelerates the growth of gastric cancer [6, 7]. Isobe et al. demonstrated that estrogen, which increases during pregnancy, promotes growth of the diffuse type of adenocarcinoma [8], or the immunosuppressive influence of pregnancy may be an additional factor in the development of the malignant process [9]. These reports indicate the possibility of the increasing risk of recurrence in pregnant cancer survivors.

When detected during pregnancy, in many cases, gastric cancer is diagnosed at an advanced stage because symptoms such as nausea and vomiting are generally overlooked during pregnancy, resulting to poor outcome [10]. Pacheco et al. [11] reported three cases of gastric cancer during pregnancy in which they were diagnosed with stage III~IV disease and all received chemotherapy with or without surgery, two of them died several years after treatment. In the present case, fortunately, the gastric cancer was detected at an early stage without lymph node metastasis and potentially curative surgery was performed. Thus, to prevent medical radiation exposure, blood examination and ultrasonography were the only routine oncologic follow-up care throughout pregnancy. More attention must be paid for patients who are diagnosed with gastric cancer at an advanced stage.

In general, strategies for the treatment of cancer in pregnancy are not different from the treatment regimens in non-pregnant women [12]; however, a woman cancer survivor who wants children might concern about the effects of adjuvant chemotherapy on future fertility or on future offspring. Bedschi et al. [13] reported that age of the patient and the type and dose of chemotherapy are the main factors determining the magnitude of the damage in the ovary. As for gastric cancer, TS-1 (tegafur, gimeracil, oteracil potassium), which is used as the standard adjuvant chemotherapy for patient with gastric cancer in Japan [14], and CDDP (cisplatin) are classified intermediate and low gonadotoxic chemotherapy respectively. However, there are limited data on fertility after any type of chemotherapy, and these data are difficult to interpret strictly [12]. For these facts, oocytes or fertilized egg cryopreservation would be better for young women, who want to have children following treatment, before receiving adjuvant chemotherapy.

It is known that gastrectomy, especially total gastric resection, caused decrease of oral intake and digestion disorders, leading to malnutrition of the patient [15]. Further, it is reported that dumping syndrome, one of the common complications of patients following gastrectomy, gets worse during pregnancy due to lower insulin sensitivity in a pregnant woman [16]. Thus, pregnant patients after gastrectomy may face more difficulty to maintain their body weight and energy intake for continuation of pregnancy and normal fetal growth. In this case, the patient needed a supplementation of iron and vitamin B_12_, which prevented anemia. This patient did not suffer from malnutrition nor dumping syndrome, and there was no need to support her. An important risk for women who underwent total gastrectomy prior to pregnancy is the malabsorption of nutrients such as vitamin B_12_, vitamin D, calcium, folate, and iron [17]. Lack of iron, vitamin B_12_, and folate can cause not only maternal complications such as severe anemia, but also fetal complications such as neural tube defects and intrauterine growth restriction, leading to vital deficiencies for the child [18]. Peck et al. [3] reported 33 cases of women who became pregnant and delivered a baby after subtotal gastrectomy for benign diseases and found anemia in 20 cases, and the supplementation of iron was administered in 11 cases and vitamin B12 in 4 cases. This suggests the possibility of increasing risk of anemia and lack of vitamins for the mother after gastrectomy. In our case, the patient was making good progress during her pregnancy with the prophylactic supplementation of iron and vitamin B_12_.

In this case, she suffered from liver dysfunction during the lactation period. One possible reason is that excessive oral caloric intake to ameliorate energy loss caused by exclusive breastfeeding was a burden to the liver function during the lactation period. According to the *Dietary Reference Intakes for Japanese (ver. 6)* created by the Ministry of Health, Labour and Welfare [19], the additional energy requirement for a pregnant or lactating woman is approximately 150 kcal/day in the early stage, 350 kcal/day in the late stage, and 700 kcal/day during lactation to maintain their current body weight and favorable neonatal growth. Dewey [20] reported additional energy needs for an exclusively breastfeeding woman are approximately 670 kcal/day. These reports suggest that energy consumption is predominantly higher during the lactation phase than during the pregnancy period and exclusively breastfeeding induces excessive energy loss of mother. To maintain the energy for exclusively breastfeeding, lots of oral intake might have been needed for the patient. On the other hand, it is demonstrated that rapid calorie intake causes free fatty acid accumulation in the liver and excessive production of ROS (reactive oxygen species), leading to liver dysfunction [21]. Thus, in the present case, it is possible that an additional formula milk provided caloric supplement to the baby and prevent energy consumption of the patients herself, contributing to the reduced amount of excessive oral intake during the lactation period and normalizing AST and ALT. Of course, there were limitations that we did not explore other variables such as her breastfeeding plan and accurate oral calorie intake during postpartum period.

## Conclusions

We report a case of a woman who became pregnant and successfully delivered a baby after total gastrectomy. During the lactation period, a combination of formula and breastfeeding provides maternal and fetal nutritional support. Careful monitoring and management of iron and vitamin deficiencies are essential during pregnancy and the lactation periods for women who previously underwent total gastrectomy.

## Abbreviations

ALT: Alanine transaminase
AST: Aspartate transaminase
CDDP: Cisplatin
LATG: Laparoscopic-assisted total gastrectomy
MeCbl: Methylcobalamin
ROS: Reactive oxygen species
TS-1: Tegafur, gimeracil, oteracil potassium
Tub2: Moderately differentiated tubular adenocarcinoma
